# Inflammation-induced osteogenic signaling promotes calcium phosphate crystal formation in kidneys via MAPK, NF-κB, and smad pathways

**DOI:** 10.3389/fcell.2026.1831072

**Published:** 2026-06-30

**Authors:** Yi-Shiou Tseng, Hsing-I Tseng, Po-Wen Ku, Huei-Pin Lai, Ting-Hui Chang, Wen-Po Chuang, Jiann-Ming Wu

**Affiliations:** 1 Division of Traumatology, Department of Surgery, Far Eastern Memorial Hospital, New Taipei, Taiwan; 2 Division of Urology, Department of Surgery, Far Eastern Memorial Hospital, New Taipei City, Taiwan; 3 Department of Chemical Engineering and Materials Science, Yuan Ze University, Taoyuan City, Taiwan; 4 Graduate Institute of Medicine, Yuan Ze University, Taoyuan City, Taiwan; 5 Department of Medical Research, Far Eastern Memorial Hospital, New Taipei, Taiwan; 6 Division of Endocrinology and Metabolism, Department of Internal Medicine, Far Eastern Memorial Hospital, New Taipei City, Taiwan; 7 Division of Allergy Immunology and Rheumatology, Department of Internal Medicine, Far Eastern Memorial Hospital, New Taipei City, Taiwan; 8 Division of Cardiology, Cardiovascular Center, Far Eastern Memorial Hospital, New Taipei City, Taiwan; 9 Division of General Surgery, Department of Surgery, Far Eastern Memorial Hospital, New Taipei City, Taiwan

**Keywords:** animal disease models, calcium phosphates, crystallization, inflammation, kidney calculi, osteogenesis

## Abstract

**Introduction:**

Approximately two-thirds of patients with kidney stones exhibit Randall’s plaques, interstitial calcium phosphate deposits extending to the papillary surface, serving as stone formation niduses. Although inflammation and osteogenic processes have been implicated in plaque development, their mechanistic connection remains unclear. In vascular calcification, which shares key pathological features with Randall’s plaques, pro-inflammatory cytokines upregulate osteogenesis-related proteins to promote calcification. Whether similar signaling occurs in kidneys remains unclear. This study aimed to explore physiopathology and facilitate the development of potential targeted therapies for kidney stones.

**Methods:**

HK-2 cells were treated with calcium and pro-inflammatory cytokines to assess calcium deposition and investigate osteogenic transition signaling pathways. Non-cell-based assays were developed to examine the direct effects of osteogenesis-related proteins on specific stages of calcium phosphate crystal formation, including nucleation, growth, and aggregation. To determine whether inflammation is related to osteogenic responses and calcification *in vivo*, a novel rat model of renal calcium deposition was established by combining a high-calcium diet with unilateral ureteral obstruction surgery. Renal calcium deposits were also evaluated.

**Results:**

*In vitro*, pro-inflammatory cytokines enhanced calcium-induced mineralization in renal tubular cells and were associated with the activation of MAPK, NF-κB, and Smad signaling pathways, along with the upregulation of osteogenesis-related proteins. These proteins exerted direct, stage-specific effects on calcium phosphate crystal nucleation, growth, and aggregation. In our novel rat model, inflammation enhanced renal calcium deposition and upregulated osteogenic markers within 1 month. The positive correlation between the renal expression of inflammatory cytokines and osteoblast markers mirrored the *in vitro* findings.

**Discussion:**

This study provides insight into renal calcification, suggesting that pro-inflammatory cytokines are associated with activation of MAPK, NF-κB, and Smad signaling pathways and upregulation of osteogenesis-related proteins, which may contribute to key stages of crystal formation. Furthermore, this work establishes a novel and time-efficient animal model that offers a valuable platform for investigating the pathogenesis of renal calcification, including processes potentially relevant to Randall’s plaque–associated calcification.

## 1 Introduction

Kidney stone disease is common, with a global prevalence of 5%–10% (Hill et al., 2022), and a recurrence rate of up to 50% within 5 years (Khan et al., 2016). The high prevalence and recurrence rates indicate that the pathological mechanisms of kidney stones must be explored to facilitate the development of novel targeted therapies (Moe et al., 2011).

Approximately 80% of kidney stones are a mixture of calcium oxalate (CaOx) and calcium phosphate (CaP) (Evan, 2010). Randall’s plaques, CaP deposits located in the renal interstitium and extending to the surface of the renal papilla, are hypothesized to serve as attachment sites for CaOx stone formation and represent the origin of stone development (Coe et al., 2005). Between 57% and 74% of patients with kidney stones exhibit Randall’s plaques, suggesting that a substantial proportion of stones form on these structures (Stoller et al., 1996; Verrier et al., 2016; Low and Stoller, 1997). Therefore, understanding the pathophysiological mechanisms underlying the formation of Randall’s plaques may provide important insights into kidney stone formation.

Both inflammatory response and osteogenesis were found to be involved in the formation of Randall’s plaque (Khan et al., 2016; Khan et al., 2021). The renal papillae of patients with Randall’s plaques demonstrated markedly increased pro-inflammatory cytokine levels relative to urinary concentrations, suggesting that the inflammatory response may be initiated in the renal papillae (Sun et al., 2018). Upregulated genes associated with inflammation, renal damage, and cellular apoptosis such as *LCN2*, *IL11*, *PTGS1*, *GPX3*, and *MMD* suggested the involvement of an inflammatory response in plaque formation (Taguchi et al., 2017). Crystals triggered ROS production and activation of the NLRP3 inflammasome, leading to the maturation of pro-inflammatory cytokines including IL-1β and IL-18, thereby building an inflammatory microenvironment that promoted plaque formation (Joshi et al., 2015).

The expression of osteoblast markers such as bone morphogenetic protein 2 (BMP-2), Runt-related transcription factor 2 (Runx2), osteopontin (OPN), and osterix increased in the kidneys of the rats with CaP deposition (Jia et al., 2014; Jia et al., 2015). Renal tubular epithelial cells from hypercalciuric rats in high-calcium conditions exhibited increased mRNA levels of osteogenic genes (Jia et al., 2015). Additionally, human renal tubular epithelial cells in osteogenic medium showed CaP deposition and upregulation of osteoblast markers such as Runx2, alkaline phosphatase (ALP), OPN, and osteonectin (Pri et al., 2019). Evidence showed that kidney tissues with Randall’s plaques exhibited significantly higher Runx2, osteocalcin, and OPN levels than those without (Zhu et al., 2020). These findings suggest a strong association between renal osteogenesis, CaP deposition, and Randall’s plaque formation.

Given the shared pathological mechanisms between Randall’s plaques and vascular calcification (Khan et al., 2016; Khan et al., 2021), along with the more advanced research on vascular calcification, it can serve as a valuable reference for further exploration into Randall’s plaques.

In vascular calcification, pro-inflammatory cytokines upregulated osteogenesis-related genes. Exposure of vascular smooth muscle cells (VSMCs) to IL-1β increased the expression of Runx2, Sry-related HMG box 9, osterix, Msh homeobox 2, and ALP through NF-κB/p53/p21 signaling pathway, leading to cell calcification (Ceneri et al., 2017; Han et al., 2020). TNF-α enhanced the binding of transcription factors associated with osteogenic differentiation, such as Osf2, AP1, and CREB, to their target genes in VSMCs. This increased the transcription of osteogenic genes and upregulated the expression of ALP and osteoprotegerin (OPG) (Tintut et al., 2000; Ben-Tal Cohen et al., 2007). TGF-β1 promoted calcification and the expression of osteogenesis-related proteins in aortic valve interstitial cells. Studies showed that both TGF-β1 and osteogenesis-related genes were upregulated in calcified tissues (Clark-Greuel et al., 2007). BMP-2, a pro-inflammatory and pro-osteogenic cytokine, increased the expression of Runx2 and OPN when administered to coronary artery smooth muscle cells (Balderman et al., 2012; Zhang et al., 2014). Pro-inflammatory cytokines may promote osteogenesis through MAPK, NF-κB, and Smad signaling pathways in vascular calcification, either directly or indirectly (Zeng et al., 2021; Liang et al., 2021; Yang et al., 2016; Beck and Knecht, 2003; Jun et al., 2010; Liu et al., 2021; Raaz et al., 2015; Liberman et al., 2011; Lee et al., 2016). However, whether similar mechanisms occur in the kidney and contribute to renal CaP deposition remains unexplored.

We found that pro-inflammatory enhanced calcium-induced mineralization and were associated with increased expression of osteogenesis-related proteins in renal tubular cells. We also established a short-term rat model for renal CaP deposition by combining a high-calcium diet with renal inflammation induced by UUO surgery, this model may provide a useful platform for studying inflammation-associated renal calcification. We found that UUO-induced inflammation exacerbated calcium deposition caused by high calcium and significantly increased the renal expression of osteoblast markers.

## 2 Materials and methods

### 2.1 Cell culture

The HK-2 cell line (BCRC Number: 60,097) was obtained from the Bioresource Collection and Research Center (BCRC, Hsinchu, Taiwan). Cells were cultured in Dulbecco’s Modified Eagle’s Medium (10–013-CM, Corning, NY, United States), supplemented with 10% fetal bovine serum (FBS) (35–010-CV-35C, Corning) and 1% penicillin-streptomycin (30–002-CI, Corning). The culture medium was changed twice a week, and cells were maintained in a 37 °C incubator with 5% CO_2_. Subculturing was performed when cell confluence exceeded 80% of the culture dish area.

### 2.2 Chemicals and recombinant proteins for *in vitro* studies

Recombinant proteins such as human TNF-α (ab9642), IL-1β (ab9617), TGF-β1 (AB50036), and BMP-2 (AB87065) were purchased from Abcam (Cambridge, MA, United States). Calcium chloride (C4901) was purchased from Merck (Darmstadt, Germany). Small molecule inhibitors such as U0126 (ERK inhibitor, 70,970), SP600125 (JNK inhibitor, 10010466), SB203580 (p38 inhibitor, 13,067), BAY 11–7,082 (NF-κB inhibitor, 10010266), SB-525334 (Smad2/3 inhibitor, 16,281), and LDN-193189 (Smad1/5/8 inhibitor, 11,802) were purchased from Cayman Chemical (Ann Arbor, MI, United States).

### 2.3 Antibodies

Primary antibodies for immunoblotting such as OPN (A19092), ALP (A0514), OPG (A2100), Runx2 (A2851), p-JNK1/2/3 (AP0631), JNK1/2/3 (A4867), p-NF-κB (AP0944), NF-κB (A19653), p-p38 (AP0057), p38 (A14401), p-ERK (AP0485), ERK (A4782), p-Smad1/5/9 (AP0850), Smad1 (A21734), p-Smad2/3 (AP0548), Smad2/3 (A7536), TNF-α (A11534), IL-1β (A11534), TGF-β1 (A16640), BMP-2 (A0231), β-actin (AC028), α-Tubulin (A6830), and GAPDH (A19056) were from ABclonal (Woburn, MA, United States). The secondary antibody Goat Anti-Rabbit IgG antibody (GTX213110-01) was purchased from GeneTex International (Hsinchu, Taiwan). Antibodies for IHC such as OPN (A1499), ALP (A0514), OPG (A2100), Runx2 (A2851), TNF-α (A11534), IL-1β (A11534), TGF-β1 (A16640), and BMP-2 (A0231) were purchased from ABclonal.

### 2.4 Alizarin red S staining

HK-2 cells in an FBS-free medium were seeded into 24-well plates at a density of 2.5 × 10^5^ cells/well. After 24 h of incubation, the indicated dosages of CaCl_2_ or cytokines (TNF-α, IL-1β, TGF-β1, or BMP-2) were applied individually or in combination for 6 days. The culture medium was removed, and the plates were washed with 1× PBS without calcium and magnesium. After PBS was removed, the cells were fixed in 10% neutral buffered formalin for 30 min. The plates were then washed with ddH_2_O, and the cells were stained with 0.02 g/mL Alizarin Red S (pH 4.1–4.3, sc-205998, Santa Cruz Biotechnology) for 45 min. After the removal of the stain, the excess stain was washed off with ddH_2_O four times.

### 2.5 CaP crystallization assay

18.2 mM CaCl_2_ (250 µL), 13.65 mM Na_2_HPO_4_ (125 µL) and 88 mM NaH_2_PO_4_ (125 µL) were mixed in each well of the 24 well-plate, and 5 µL of different concentrations of recombinant ALP, OPG or OPN were added. After incubation at 37 °C for 60 min, the supernatant was carefully removed. 300 μL of 2% alizarin red solution (pH = 4.2) was added to each well of the plate and incubated at 37 °C for 15min. The solution and the stained crystals were collected and the plate was washed by ddH2O 3 times. Centrifuge at 10,000 g for 20min and the supernatant was discarded. Alizarin red was extracted from the stained crystals with 10% acetic acid, incubated at room temperature with shaking for 30 min, and then heated in a dry bath at 85 °C for 10 min. The pH of the solution was adjusted to approximately 4.2 using KOH. Samples were diluted 1:3 with ddH2O and absorbance was read at 405 nm (BioTek Epoch 2, Agilent, Santa Clara, CA, United States). Alizarin red staining is a well-known method to detect the presence of CaP crystals and quantify the mineralization.

### 2.6 CaP growth assay

18.2 mM CaCl_2_ (250 µL), 13.65 mM Na_2_HPO_4_ (125 µL) and 88 mM NaH_2_PO_4_ (125 µL) were mixed in each well of the 24-well plate. The CaP crystals were formed after incubation at 37 °C for 60 min. Crystal images were captured using an inverted light microscope equipped with a digital camera (this time point was set as T0). 5 μL of different concentrations of ALP, OPG or OPN were added to the wells and incubated at 37 °C for another 60 min. At this time point (T60), crystal images were again captured. Crystal growth was reported as Δcrystal size, which was calculated by the formula: ΔCrystal size (μm^2^) = Crystal size at T60 - Crystal size at T0. Crystal sizes were calculated from 5 fields per condition and were analyzed by ImageJ.

### 2.7 CaP aggregation assay

18.2 mM CaCl_2_ (500 µL), 13.65 mM Na_2_HPO_4_ (250 µL) and 88 mM NaH_2_PO_4_ (250 µL) were mixed and stirred at RT for 3 min 10 μL of different concentrations of ALP, OPG or OPN were added. The solution was stirred at RT for an additional 60 min. Following this, 500 µL of the mixture was carefully transferred onto microscope slides with coverslips, ensuring the crystals formed a thin, uniform layer. Images of the aggregated crystals were captured using an inverted light microscope equipped with a digital camera. CaP aggregates were defined as “clusters consisting of at least three individual crystals adhered together”. The number and area of the aggregates were counted from 3 fields per condition and were analyzed by ImageJ. The average area per aggregate was calculated by the formula: The total area of the aggregates in each field/the number of the aggregates in each field.

### 2.8 Animal studies

5-week-old male Sprague Dawley (SD) rats were obtained from Lasco (Taipei, Taiwan). This age group was selected to establish a stable renal baseline and minimize potential confounding effects from spontaneous age-related renal lesions, including chronic progressive nephropathy, which has been reported to begin developing in male SD rats from approximately 8–12 weeks of age (Hard and Khan, 2004; Hard et al., 2013). Rats were divided into four groups: control (n = 3), high-calcium diet (Ca group, n = 3), unilateral ureteral obstruction surgery (UUO group, n = 6), and high-calcium diet combined with UUO (UUO + Ca group, n = 8). Control rats were fed a normal diet and underwent sham surgery on the first day. Ca rats were administered Calcium D-gluconate (2 g/L, sc-221393, Santa Cruz Biotechnology) in their drinking water for 28 days and received vitamin D3 (1 × 10^5^ IU/kg, i. p.) once per week. UUO rats were fed a normal diet and underwent UUO surgery on the left ureter on the first day. UUO + Ca rats were given a high-calcium diet and vitamin D3 (1 × 10^5^ IU/kg/week i. p.) for 28 days, with UUO surgery performed on the left ureter on the first day. We used a combined metabolic and renal injury-induced model to induce renal CaP deposition under inflammatory stress conditions. All animal experiments were performed in accordance with relevant guidelines and regulations following ethical standards, and protocols were reviewed and approved by the Animal Use and Management Committee of Far Eastern Memorial Hospital (IACUC number: IACUC-2021-FYM-04). The animals were maintained in standard cages and conditions (temperature: 22 °C ± 1 °C, light-dark cycle: 12 h, humidity: 50%–60%). They had unlimited access to drinking water and commercial laboratory chow. Two rats were housed in one cage and were cared for every day.

### 2.9 24-h urine, plasma, and kidney collection

One day before sacrifice, the rats were placed in metabolic cages for 24-h urine collection. On the day of sacrifice, the animals were anesthetized via intraperitoneal injection of Zoletil 50 (1 mL/kg). Blood was collected from the heart and centrifuged at 3,000×g at room temperature for 10 min to separate the plasma. The collected plasma and urine were stored at −80 °C for biochemical analyses. The right kidneys were removed, washed with ice-cold saline, and blotted dry with filter paper. A small portion of the kidney was fixed in 10% neutral buffered formalin and embedded in paraffin using standard techniques performed by the National Laboratory Animal Center (Taipei, Taiwan) for histological analysis. The remaining kidney tissue was stored at −80 °C for molecular analysis.

### 2.10 Biochemical tests for plasma and 24-h urine

Concentrations of plasma BUN and creatinine were measured by the Taipei Institute of Pathology. Urinary calcium, sodium, magnesium, phosphorus, potassium, and creatinine were also analyzed by the Taipei Institute of Pathology. Urinary pH was determined using a pH meter. Urinary oxalate was analyzed using the Oxalate Assay Kit (EOXA-100, BioAssay Systems, Hayward, CA, United States) according to the manufacturer’s instructions. Urine samples were acidified by adding 6N HCl (0.02% w/w) before being stored at −80 °C. Before oxalate determination, the samples were neutralized by mixing with 6N NaOH and centrifuged at 13,000×g at 4 °C for 5 min to collect the supernatant for analysis. Urinary citrate was analyzed using the Citrate Assay Kit (ab83396, Abcam, Cambridge, MA, United States) according to the manufacturer’s protocol. Urine samples were deproteinized by adding ice-cold 4M PCA to a final concentration of 1M and neutralized to pH 6.5–8 by mixing with ice-cold 2M KOH. The samples were then centrifuged at 13,000×g for 15 min at 4 °C, and the supernatant was collected for the assay. The degree of urine supersaturation was calculated according to the formula of the ion activity product of CaOx (AP(CaOx)) and CaP (AP(CaP)) as follows:

$$\text{AP}\left(\text{CaOx}\right)=\frac{4076\times {\text{Calcium}}^{0.93}\times {\text{Oxalate}}^{0.96}}{{\left(\text{Citrate}+0.015\right)}^{0.60}\times {\text{Magnesium}}^{0.55}\times {\text{Volume}}^{0.99}}$$

$$\text{AP}\left(\text{CaP}\right)=\frac{2.7\times 10^{-3}{\times \text{Calcium}}^{1.07}\times {\text{Phosphorus}}^{0.70}{\times \left(\text{pH}-4.5\right)}^{6.8}}{{\text{Citrate}}^{0.20}\times {\text{Volume}}^{1.31}}$$

### 2.11 Western blotting

The treated cells and rat tissues were lysed using RIPA Lysis Buffer (SC-24948, Santa Cruz Biotechnology, Paso Robles, CA, United States) supplemented with protease and phosphatase inhibitors. Protein concentrations were determined using the BCA Protein Assay Kit (JB04-D001, T-Pro Biotechnology, New Taipei, Taiwan). Equal amounts of total protein (30 μg) were separated by SDS-PAGE and transferred onto Immobilon-E PVDF membranes (IEVH00005, Merck Millipore, Darmstadt, Germany). The membranes were blocked with 3% (w/v) BSA for 1 h at room temperature and then incubated with primary antibodies overnight at 4 °C. Secondary antibodies were applied at RT for 1 h. Protein bands were detected using a chemiluminescence system (JT96-K004M, T-Pro Biotechnology) and visualized on a luminescence/fluorescence imaging system (LAS-4000, ImageQuant™, GE Healthcare, Chicago, IL, United States). Western blotting was performed at least three times, and quantification was done using ImageJ software (NIH, Bethesda, MD, United States). GAPDH, β-actin, or tubulin served as loading controls.

### 2.12 Histological analysis

For H&E staining, paraffin-embedded tissues were cut into 5 µm-thick sections and stained with H&E according to standard procedures performed by the National Laboratory Animal Center. Von Kossa staining, used for histological visualization of calcium deposits, was also performed by the National Laboratory Animal Center. Immunohistochemical analysis was performed using CRF Anti-Polyvalent HRP Polymer (DAB) Stain Kit (STK-CPH080, ScyTek, Logan, UT, United States) according to the manufacturer’s instructions. In brief, paraffin sections were conducted with antigen retrieval and incubated with Peroxide Block for 15 min at room temperature. After washing with PBS (pH 7.42), the samples were blocked with Super Block for 5 min and then incubated with the primary antibody (1:400) overnight at 4 °C. Following three rinses in PBS, the samples were incubated with CRF Anti-Polyvalent HRP (secondary antibody) for 30 min at room temperature. Tissue staining was visualized using a DAB Chromogen/Substrate mixture. The slides were then counterstained with hematoxylin (STK-HAQ500, ScyTek) for 3 min, dehydrated, and mounted.

### 2.13 Composition analysis of calcium deposition by FTIR spectroscopy

The renal tissues of the rats were analyzed using FTIR spectroscopy (Nicolet iN10, ThermoFisher, Waltham, MA, United States) in the frequency range of 700–4,000 cm^−1^ and at 4 cm^−1^ resolutions. CaP powder was used as the standard control substance. The composition of the calcium deposition was determined by comparison with standard control spectra.

### 2.14 Statistical analysis

Data are presented as mean ± SD or SEM for *invitro* and *invivo* studies. Significant differences between means were determined using Student’s t-test or one-way ANOVA with Tukey’s HSD test using GraphPad Prism (GraphPad Software, La Jolla, CA, United States; Version 6). Statistics are included in the figure legends. *P < 0.05, **P < 0.01, ***P < 0.001.

## 3 Results

### 3.1 Inflammation aggravates calcium-induced calcification in human renal tubular cells

**FIGURE 1:**
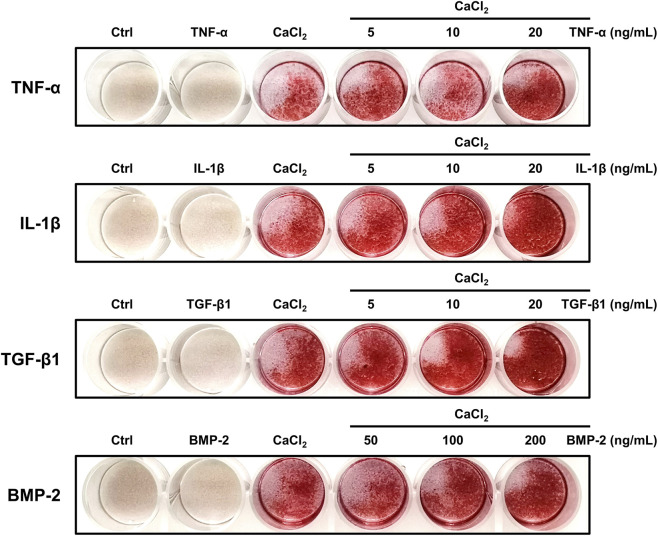
Cytokines Enhanced the Calcification of HK-2 Cells in High Calcium Environment. HK-2 cells were stimulated with CaCl_2_ (10 mM) combined with TNF-α, IL-1β, TGF-β1 (5, 10, 20 ng/mL) or BMP-2 (50, 100, 200 ng/mL) for 6 days. Alizarin red S staining was performed to observe the degree of cellular calcium mineralization.

Given the involvement of inflammation in CaP renal calcification, we examined whether pro-inflammatory cytokines exacerbate calcification induced by high calcium in human renal tubular epithelial cells (HK-2). Cells were treated with calcium chloride (CaCl_2_) alone or in combination with cytokines including TNF-α, IL-1β, TGF-β1, and BMP-2 for 6 days. Alizarin Red S staining, which detects calcium deposition through its binding to calcium ions to form an orange-red complex, was used to visualize mineralization. Although cytokines alone did not induce mineralization in HK-2 cells, they had an additive effect on calcification induced by high calcium (Figure 1). These findings suggested that immune activity amplified calcium-induced calcification in renal tubular cells.

### 3.2 Calcium and inflammatory cytokines induce osteogenic transition in renal cells

**FIGURE 2:**
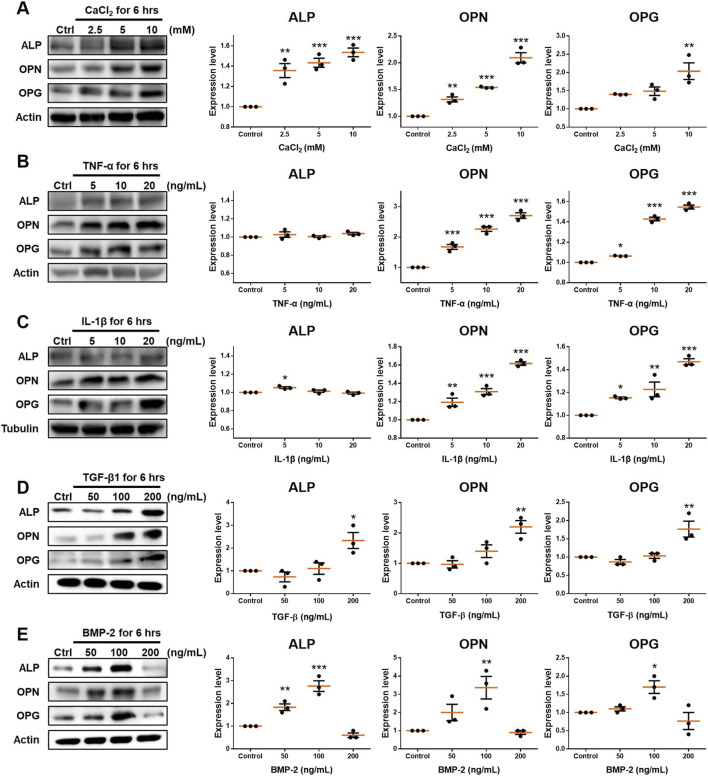
High Calcium and Pro-inflammatory Cytokines Increased the Levels of Osteogenesis-Related Proteins in HK-2 Cells. Western blot analysis of ALP, OPN, and OPG in HK-2 cells treated with CaCl_2_ **(A)** TNF-α **(B)** IL-1β **(C)** TGF-β1 **(D)** or BMP-2 **(E)** for 6 h. The quantitative results showed the fold change of the protein expression compared to the control group. β-actin or tubulin served as the loading control. Data are presented as individual data points (scatter plot) with the mean ± SEM; n = 3; *P < 0.05, **P < 0.01, ***P < 0.001 versus the control group (One-way ANOVA).

Primary culture of renal tubular epithelial cells from hypercalciuric rats in high calcium significantly elevated the mRNA expression of osteogenesis-related genes (Jia et al., 2015). To assess a similar response in human cells, HK-2 cells were treated with increasing CaCl_2_ concentrations (2.5–10 mM) for 6 h. Calcium dose-dependently upregulated osteogenic proteins, including ALP, OPN, and OPG, consistent with previous findings (Figure 2A).

Given that IL-1β promotes osteogenic gene expression in vascular calcification (Ceneri et al., 2017; Han et al., 2020; Tintut et al., 2000; Ben-Tal Cohen et al., 2007; Clark-Greuel et al., 2007; Balderman et al., 2012; Zhang et al., 2014), we hypothesized that a similar mechanism might be involved in CaP deposition in renal tubular epithelial cells. HK-2 cells were stimulated with TNF-α, IL-1β, TGF-β1, or BMP-2 for 6 h. All cytokines significantly increased the expression of osteoblast markers such as OPN and OPG, supporting the role of inflammation in promoting osteogenic transition in renal tubular epithelial cells (Figures 2B–E).

### 3.3 Cytokines upregulate osteogenic proteins and are associated with MAPK and smad signaling

**FIGURE 3:**
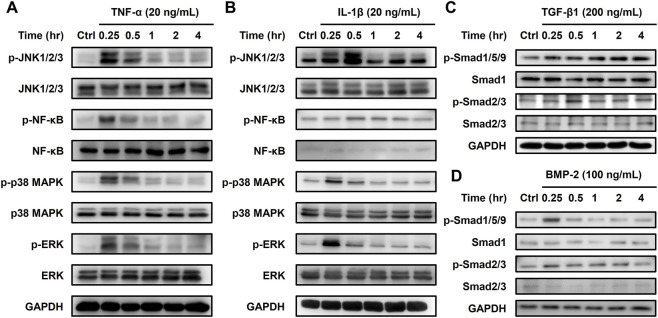
Cytokines Activated Their Downstream Pathways in HK-2 Cells. The phosphorylation of ERK, JNK, p38, and NF-κB in HK-2 cells treated with TNF-α **(A)** or IL-1β **(B)** was analyzed over a time course ranging from 15 min to 4 h. The phosphorylation of Smad1/5/9 and Smad2/3 in HK-2 cells treated with TGF-β1 **(C)** or BMP-2 **(D)** was analyzed over a time course ranging from 15 min to 4 h. GAPDH was served as the loading control.

**FIGURE 4:**
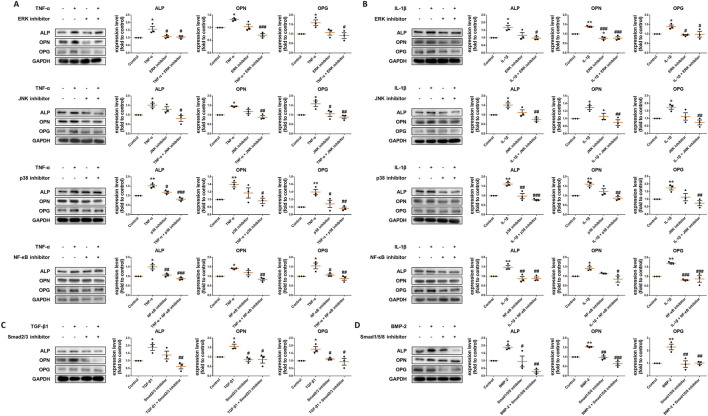
Pro-inflammatory Cytokines Upregulated Osteogenesis-related Proteins via the MAPK/NF-κB and Smad Signaling Pathway. HK-2 cells were pretreated with 10 µM ERK inhibitor (U0126), JNK inhibitor (SP600125), p38 inhibitor (SB203580), or NF-κB inhibitor (BAY 11–7,082) for 2 h, and then treated with 20 ng/mL TNF-α **(A)** or IL-1β **(B)** for 72 h **(C)** HK-2 cells were pretreated with 5 µM Smad2/3 inhibitor (SB-525334) for 2 h and then treated with 200 ng/mL TGF-β1 for 72 h **(D)** HK-2 cells were pretreated with 10 µM Smad1/5/8 inhibitor (LDN-193189) for 2 h and then treated with 100 ng/mL BMP-2 for 72 h. The expression of osteoblast markers (ALP, OPN, and OPG) was examined by Western blotting. The quantitative results showed the fold change of the protein expression compared to the control group. GAPDH served as the loading control. Data are presented as individual data points (scatter plot) with the mean ± SEM; n = 3; *P < 0.05, **P < 0.01, ***P < 0.001 versus the control group; ^#^ *P <* 0.05, ^##^ *P <* 0.01, ^###^ *P <* 0.001 versus cytokine group (One-way ANOVA).

To elucidate the molecular mechanisms through which pro-inflammatory cytokines induce osteogenic protein expression, we examined key signaling pathways known to mediate cytokine-driven osteogenesis in vascular calcification. We first confirmed that IL-1β, TNF-α, TGF-β1, and BMP-2 activated their respective downstream signaling pathways in HK-2 cells (Figure 3). To assess functional involvement, cells were pretreated with specific inhibitors targeting ERK, JNK, p38, or NF-κB prior to IL-1β or TNF-α stimulation, and with Smad2/3 or Smad1/5/8 inhibitors prior to TGF-β1 or BMP-2 treatment. Inhibition of these pathways attenuated cytokine-induced osteogenic protein expression, indicating their potential involvement. (Figure 4).

### 3.4 Osteogenesis-related proteins influence CaP stone formation *in vitro*

Kidney stone formation involves four sequential processes: crystal nucleation, growth, aggregation, and adhesion within renal structures. Although well-established for CaOx stones, comprehensive methods to study CaP stone formation remain limited. We established *in vitro* assays to investigate direct effects of osteogenesis-related proteins on stages of CaP stone formation.

Test concentrations of osteogenesis-related proteins were selected based on their physiological levels in human urine. Urinary OPG levels typically range from 0 to 500 pg/mL, with elevated concentrations observed in individuals with diabetes or nephrolithiasis (Nehru et al., 2024; Dayapule et al., 2021); thus, we tested up to 1,000 pg/mL. Urinary OPN levels typically range from 0 to 500 ng/mL and may rise in kidney disease. Urinary OPN concentrations typically range from 0–500 ng/mL and may rise in kidney disease (Moszczuk et al., 2022; Feldreich et al., 2017); hence, a concentration of up to 1,000 ng/mL was applied.

**FIGURE 5:**
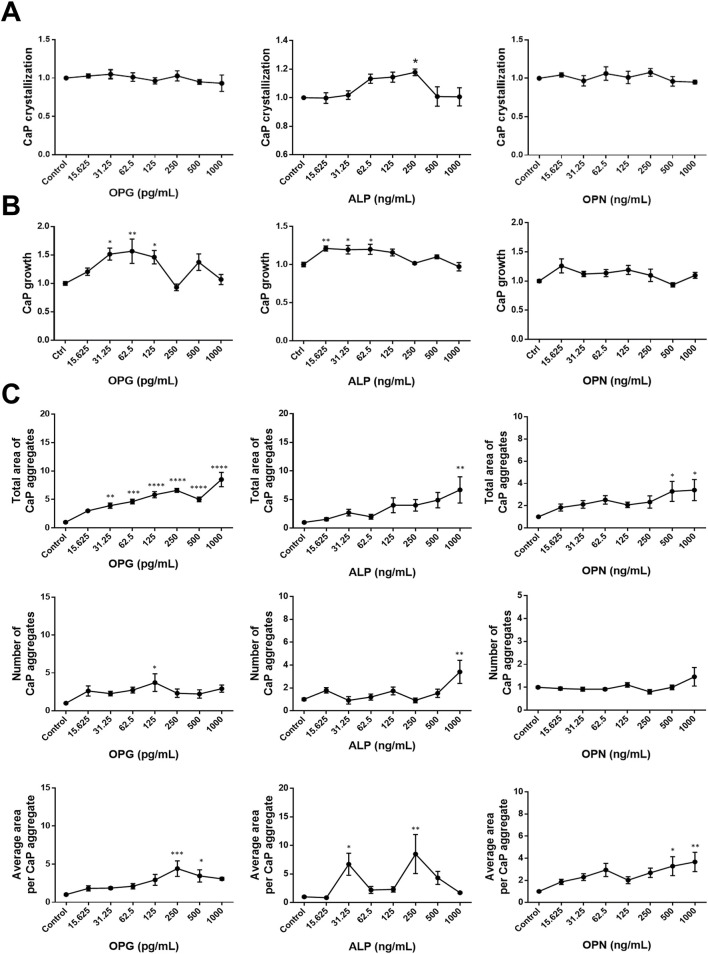
Osteogenesis-related Proteins Directly Regulate Crystal Nucleation, Growth, and Aggregation. **(A)** CaP crystallization in the presence of OPG, ALP, or OPN was quantified by alizarin red staining. Alizarin red was extracted from the stained crystal and the absorbance was read at 405 nm. The quantitative results showed the fold change of the absorbance compared to the control group. **(B)** CaP crystals were incubated with OPG, ALP, or OPN for 60 min, the change in the crystal size indicated the crystal growth. The quantitative results indicated the fold change in crystal growth of the experimental group compared to that of the control group. **(C)** CaP crystals were stirred with ALP, OPN or OPG for 60 min, the number and area of the aggregates were counted. The average area per aggregate was calculated by the formula: The total area of the aggregates in each field/the number of the aggregates in each field. The quantitative results showed the fold change to the control group. Data are mean ± SEM; *P < 0.05, **P < 0.01, ***P < 0.001, ****P < 0.0001 versus the control group (one-way ANOVA, n = 3).

ALP significantly promoted CaP nucleation and crystallization, while OPG and OPN had minimal effects (Figure 5A). ALP and OPG increased crystal size, whereas OPN had minimal impact (Figure 5B).

In aggregation assays, OPG increased the total area and the number of CaP aggregates, with a pronounced effect on individual aggregate size. ALP significantly increased the total area and number of CaP aggregates but had no impact on individual aggregate sizes at the highest tested concentration (1,000 ng/mL). Conversely, OPN increased the total area and size of the aggregates but did not affect their number. OPN and OPG mainly promoted larger aggregates (>10 CaP crystals), while ALP favored more numerous smaller aggregates (3–5 crystals). These findings suggested that all three osteogenesis-related proteins enhance CaP crystal aggregation through distinct mechanisms and modes of action (Figure 5C).

These non-cell-based assays suggest that the three osteogenic proteins directly influence CaP stone formation, each affecting different stages via distinct mechanisms and varying impact.

### 3.5 Inflammation aggravates CaP deposition in a rat model

As inflammatory activation enhanced calcium-induced calcification in HK-2 cells, we investigated its potentia*l in vivo* contribution to renal CaP deposition. We also aimed to shorten the conventional timeframe of CaP deposition in rats induced by vitamin D and a high-calcium diet (Letavernier et al., 2016), by introducing renal inflammatory stress via unilateral ureteral obstruction (UUO).

Sprague Dawley (SD) rats were divided into four groups: control, high-calcium diet, UUO, and high-calcium diet plus UUO. The UUO + Ca group received a high-calcium diet and vitamin D for 28 days, with UUO surgery performed on day 1. UUO was conducted on the left ureter, and we hypothesized that it would induce renal inflammatory stress and may influence CaP deposition in the contralateral kidney. On day 29, 24-h urine, plasma, and right kidney tissues were collected for analysis.

Biochemical assessments (Table 1) revealed significantly increased urinary calcium excretion in both Ca and UUO + Ca groups compared to controls. Although urinary sodium, potassium, magnesium, and phosphorus levels were elevated in the Ca group, their 24-h excretion did not differ significantly across groups. Citrate excretion was reduced in Ca and UUO + Ca rats, consistent with hypocitraturia as a lithogenic risk factor (Khan et al., 2021). Ion activity product of CaOx (AP(CaOx) index) and CaP (AP(CaP) index) were calculated to estimate urine supersaturation. AP(CaOx) was modestly elevated in the Ca and UUO + Ca groups, likely due to increased urinary calcium and decreased citrate rather than oxalate, which showed no group differences. AP(CaP) was significantly elevated in the Ca group and mildly elevated in the UUO + Ca group. Plasma BUN was elevated in the UUO and UUO + Ca groups, with UUO + Ca rats exhibiting worse creatinine clearance, indicating UUO-induced renal dysfunction.

**TABLE 1:** Biochemical and physiological parameters of 24-h urine and plasma, and body weight.

Parameters	​	Ctrl	Ca	UUO	UUO + Ca
24-h urine					
Urine volume (mL)		42.50 ± 10.61	25.00 ± 0.00	38.00 ± 11.26	32.00 ± 10.57
pH		6.87 ± 0.02	6.22 ± 0.08**	6.57 ± 0.29	6.28 ± 0.57
Ca	Conc. (mmol/L)	0.29 ± 0.12	9.33 ± 3.68*	0.42 ± 0.1	6.14 ± 3.93
	Excretion (mmol)	0.01 ± 0.01	0.23 ± 0.09*	0.02 ± 0.01	0.17 ± 0.09*
Na	Conc. (mmol/L)	23.50 ± 4.95	39.00 ± 1.00*	23.17 ± 5.38	25.50 ± 7.71^†^
	Excretion (mmol)	0.97 ± 0.04	0.98 ± 0.03	0.85 ± 0.19	0.81 ± 0.35
K	Conc. (mmol/L)	47.30 ± 13.58	96.17 ± 14.76*	68.07 ± 25.96	66.95 ± 35.88
	Excretion (mmol)	1.94 ± 0.08	2.40 ± 0.37	2.38 ± 0.24	1.98 ± 0.75
Mg	Conc. (mmol/L)	4.50 ± 0.20	11.52 ± 2.11*	6.80 ± 3.19	7.15 ± 3.94
	Excretion (mmol)	0.19 ± 0.06	0.29 ± 0.05	0.23 ± 0.04	0.21 ± 0.09
P	Conc. (mmol/L)	19.96 ± 6.03	39.62 ± 6.3*	26.36 ± 12.98	23.7 ± 13.95
	Excretion (mmol)	0.82 ± 0.04	0.99 ± 0.16	0.89 ± 0.15	0.68 ± 0.27
Creatinine	Conc. (μmol/L)	2466.36 ± 725.10	4396.43 ± 274.23*	2529.71 ± 954.06	2148.12 ± 1138.35^††^
	Excretion (μmol)	100.97 ± 4.66	109.91 ± 6.86	88.32 ± 7.72	63.93 ± 24.92^†^
Oxalate	Conc. (µmol/L)	654.77 ± 190.59	866.82 ± 563.52	575.12 ± 269.95	657.54 ± 684.1
	Excretion (µmol)	26.82 ± 1.15	21.67 ± 14.09	20.36 ± 8.34	22.03 ± 26.51
Citrate	Conc. (µmol/L)	40.75 ± 0.08	33.13 ± 4.32	36.44 ± 6.82	26.30 ± 6.48*
	Excretion (µmol)	1.73 ± 0.42	0.83 ± 0.11*	1.35 ± 0.35	0.88 ± 0.45*
Saturation index	AP (CaOx)	1.44 ± 0.26	25.71 ± 16.34	1.58 ± 1.01	17.72 ± 23.75
	AP (CaP)	1.62 ± 0.40	11.53 ± 2.88*	1.46 ± 1.05	6.98 ± 8.22
Plasma					
BUN (mmol/L)		5.50 ± 0.81	5.13 ± 0.54	6.89 ± 0.52*	9.73 ± 4.84
Creatinine (μmol/L)		54.81 ± 5.00	51.57 ± 7.41	49.5 ± 2.25	51.05 ± 8.22
Creatinine clearance (mL/minute)		1.28 ± 0.06	1.50 ± 0.22	1.24 ± 0.15	0.89 ± 0.36†
Body weight					
Initial (g)		164.55 ± 0.21	168.50 ± 0.99	130.20 ± 6.10	140.38 ± 3.91
Final (g)		299.50 ± 4.95	315.00 ± 2.83	288.00 ± 12.87	233.38 ± 62.18

Data are mean ± SD; *P < 0.05, **P < 0.01 versus the control group; †*P* < 0.05, ††*P* < 0.01 versus Ca group (unpaired t-tests). Conc., concentration.

**FIGURE 6:**
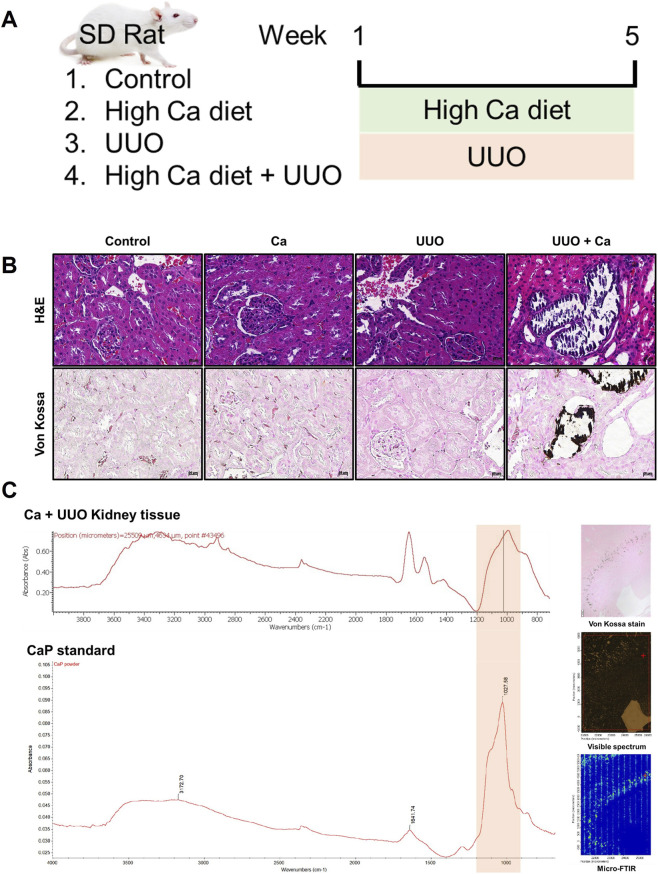
UUO Increased Renal Deposition in Rats Fed a High Calcium Diet. **(A)** Control rats were fed a normal diet and had sham surgery on the first day. Calcium gluconate (2 g/L) was administered to Ca rats in drinking water for 4 weeks. Ca + UUO rats were fed a high-calcium diet for 4 weeks and had UUO surgery on the first day. **(B)** Hematoxylin and eosin (H&E) staining of rat kidney tissues (*top*, 400×). Renal CaP deposition was examined by Von Kossa staining (*bottom*, 400×). **(C)** Tissue components measured by Micro-FTIR. The distribution position of CaP measured by Micro-FTIR (*Right*, *bottom*) is consistent with the Von Kossa stain (*Right*, *top*).

Histological analysis (H&E and Von Kossa staining, Figure 6B) showed no detectable calcium deposits in the Ca group despite increased urinary supersaturation. In contrast, CaP deposits were observed in the UUO + Ca group, accompanied by histological signs of renal tissue injury (Figure 6B; Supplementary Figure 1). Micro-Fourier Transform Infrared Spectroscopy (micro-FTIR) confirmed that the deposits exhibited characteristic CaP absorption peaks (900–1,200 cm^-1^) (Figure 6C), consistent with Von Kossa-positive regions.

Collectively, UUO-induced inflammatory stress was associated with increased renal CaP deposition under high-calcium conditions.

### 3.6 *In vivo* upregulation of osteogenesis-related proteins by inflammation

**FIGURE 7:**
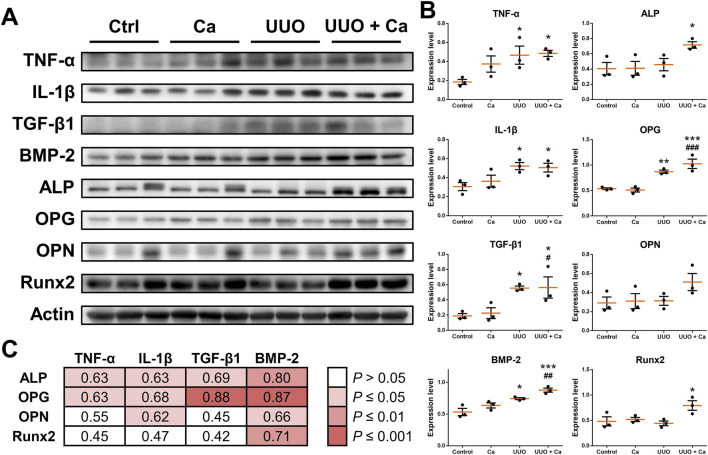
Inflammation Positively Correlates with Osteogenesis in Renal CaP Deposition. **(A)** Western blot analysis of expression of inflammatory cytokines and osteoblast markers in the kidney tissues of the rats. β-actin served as the loading control. **(B)** The quantitative results showed the protein level compared to the loading control. Data are presented as individual data points (scatter plot) with the mean ± SEM; n = 3; *P < 0.05, **P < 0.01, ***P < 0.001 versus the control group; ^#^ *P <* 0.05, ^##^ *P <* 0.01, ^###^ *P <* 0.001 versus Ca group (One-way ANOVA). **(C)** Pearson correlation coefficients (r) and *P*-values (*P*) were calculated for each pair of osteoblast marker levels and inflammatory cytokine levels. The results are presented as a heatmap. Data are Pearson correlation coefficients (r) with the colors indicating the corresponding *P*-values (*P*).

**FIGURE 8:**
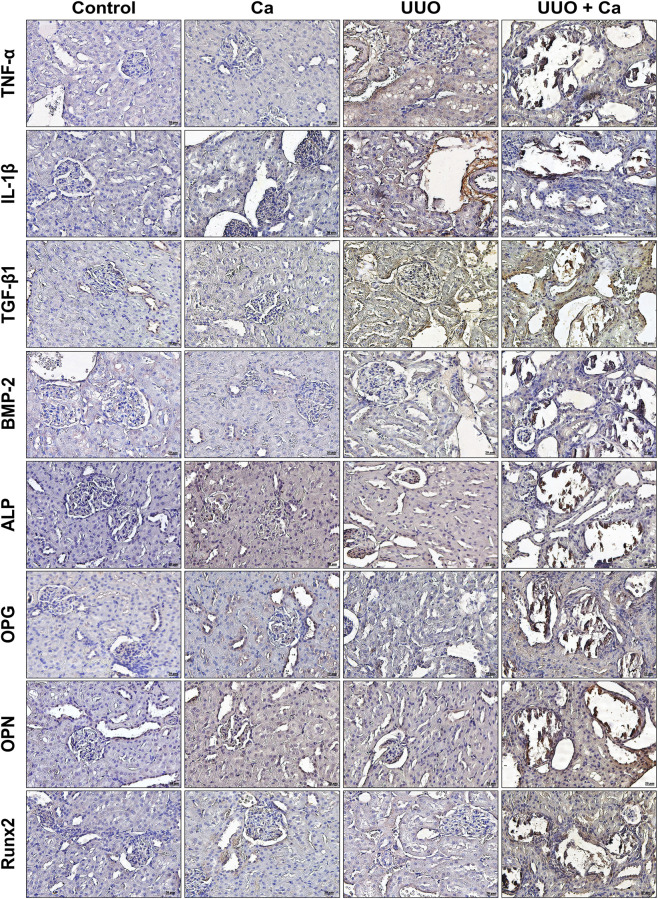
Representative Immunohistochemical Staining of Inflammatory Cytokines and Osteoblast Markers in the Kidneys of Rats Fed a High-Calcium Diet. Representative images of TNF-α, IL-1β, TGF-β1, BMP-2 (inflammatory cytokines) and OPN, OPG, ALP, Runx2 (osteogenesis-related proteins) in kidney tissues from each experimental group are shown (400×). IHC staining was performed to visualize the spatial distribution of target proteins within renal tissue. Scale bar, 20 µm.

To further investigate the association between inflammation and osteogenesis-related changes *in vivo*, we analyzed the expression of inflammatory cytokines and osteogenesis-related proteins via Western blotting and immunohistochemistry (Figures 7, 8). TNF-α, IL-1β, TGF-β1, and BMP-2 were upregulated in the kidney tissues of UUO and UUO + Ca rats, as shown by Western blot analysis, indicating increased inflammatory cytokine expression associated with UUO. A high-calcium diet alone did not significantly alter levels of osteoblast markers such as ALP, OPG, OPN, and Runx2. In contrast, the combination of UUO and high-calcium diet was associated with increased expression of these proteins compared with the Ca group (Figure 7). Notably, immunohistochemistry provided representative spatial evidence of protein distribution (Figure 8). Representative IHC staining showed more prominent signals for these markers in the UUO and UUO + Ca groups, with osteogenic marker-positive regions colocalizing with calcium deposits shown by Von Kossa staining in Figure 6B. Correlation analysis based on protein quantification (Western blot) demonstrated positive associations between inflammatory cytokines and osteogenesis-related markers (correlation coefficients = 0.42–0.88), supporting a link between inflammatory activity and osteogenic-like changes in renal tissue. These findings are consistent with our *in vitro* results, suggesting that inflammatory activity may contribute to osteogenesis-related protein expression in renal tubular cells under calcium stress conditions.

## 4 Discussion

Patients with Randall’s plaques often exhibit elevated levels of inflammatory cytokines and osteogenesis-related proteins in renal tissues, suggesting a potential link between inflammation and osteogenesis in calcium-based stone formation. Our findings suggest at the cellular and molecular levels that inflammatory responses may be associated with osteogenic-like changes in renal tubular cells, thereby contributing to renal calcification and potentially to processes relevant to Randall’s plaque development. Furthermore, we provide evidence suggesting that this process may involve the upregulation of osteogenesis-related proteins associated with renal calcification, potentially involving MAPK, NF-κB, and Smad signaling pathways.

We demonstrated that pro-inflammatory cytokines synergize with high calcium to enhance calcification in human renal tubular cells. In an inflammatory microenvironment simulated by TNF-α, IL-1β, TGF-β1, and BMP-2, calcium-induced mineralization was markedly amplified (Figure 1). We also established a short-term rat model of renal CaP deposition by combining a high-calcium diet with UUO-induced inflammation (Figure 6). Together, the *in vitro* and *in vivo* findings suggest that inflammatory activation is associated with increased renal CaP deposition under high-calcium conditions, consistent with observation*s* in vascular calcification (Ceneri et al., 2017; Han et al., 2020; Tintut et al., 2000; Ben-Tal Cohen et al., 2007; Clark-Greuel et al., 2007; Balderman et al., 2012; Zhang et al., 2014).

Our data confirmed that cytokines significantly increased the expression of osteogenesis-related proteins including ALP, OPN, and OPG, supporting the possibility of osteogenic-like phenotypic changes in tubular epithelial cells (Figure 2). In our UUO + Ca rat model, elevated levels of both inflammatory cytokines and osteogenic markers were observed. Their positive correlation suggests an association between inflammatory activation and osteogenesis-related responses in renal tissue (Figures 7, 8). These *in vivo* findings corroborate our *in vitro* results and highlight the potential role of inflammation-driven osteogenic signaling in renal CaP deposition, including processes that may be relevant to Randall’s plaque formation.

As mentioned above, we observed that cytokine-induced upregulation of osteogenesis-related proteins was associated with MAPK, NF-κB, and Smad pathway activity *in vitro*. Inhibition of these pathways attenuated the cytokine-induced upregulation of osteogenesis markers (Figure 4). These findings are consistent with observations in vascular calcification, where these pathways have been implicated in the regulation of Runx2 and osteogenic gene expression. For example, ERK expression correlates with Runx2 and calcification severity in aortic valves, and ERK inhibition reduces OPN, ALP, and Runx2 in vascular smooth muscle cells (VSMCs) (Zeng et al., 2021; Liang et al., 2021; Yang et al., 2016; Beck and Knecht, 2003). ERK also stabilizes Runx2 and enhances its transcriptional activity (Jun et al., 2010). Similarly, p38 and JNK increase Runx2 activity and osteogenic protein expression, while their inhibition downregulates ALP, osteocalcin, and OPN (Liu et al., 2021; Yang et al., 2018; Chang et al., 2020; Koike et al., 2016). NF-κB enhances Runx2 transcription by binding its promoter (Raaz et al., 2015). BMP-2 promotes calcification via Smad1/5/8-mediated upregulation of Runx2 (Liberman et al., 2011; Lee et al., 2016). These findings suggest that similar signaling pathways may be involved in calcification processes across different tissues. Importantly, our *in vitro* data identify MAPK, NF-κB, and Smad signaling as candidate pathways involved in cytokine-induced osteogenic responses based on pharmacological modulation. While these findings support their functional participation, definitive validation of pathway specificity and causal necessity will require more targeted genetic approaches, such as siRNA-mediated gene silencing or pathway-specific *in vivo* blockade.

The lack of a suitable and accessible animal model that faithfully recapitulates Randall’s plaque has long hindered mechanistic research in this field (Daudon et al., 2015; Chidambaram et al., 2015). The most commonly used model, genetic hypercalciuric rats developed through selective inbreeding over 29 generations, exhibit pronounced hypercalciuria, but CaP deposits primarily localize within renal tubules rather than the interstitium (Bushins et al., 1995), failing to replicate key pathological features of human Randall’s plaque. Similarly, Spp1^−/−^ mice develop interstitial CaP deposits in only ∼10% of cases (Wu, 2015), while Npt2a^−/−^ mice display mixed tubular and interstitial deposition without subsequent CaOx overgrowth on CaP, lacking critical features associated with plaque progression (Khan et al., 2008). Although Abcc6^−/−^ mice form CaP at the basement membrane of Henle’s loop, resembling certain aspects of human disease (Letavernier et al., 2018), the high cost and technical demands of knockout models restrict their widespread use. Alternatively, long-term exposure (6 months) to a high-calcium diet and vitamin D can induce CaP deposition in the renal papillae (Letavernier et al., 2016), although the extended timeframe constrains experimental flexibility. Collectively, these models have provided important insights into renal mineralization but are primarily driven by altered ion transport or deficiency of endogenous crystallization inhibitors, and are not specifically designed to capture inflammation-associated mechanisms that may be relevant to renal calcification and Randall’s plaque formation.

Rather than attempting to fully replicate the complex histological architecture of human Randall’s plaque, the present study focuses on modeling renal CaP deposition under combined inflammatory and metabolic stress conditions, representing early pathogenic processes potentially relevant to calcification associated with Randall’s plaques. In this context, we developed a practical and time-efficient (one-month) rat model of renal CaP deposition by combining a high-calcium diet, vitamin D supplementation, and UUO-induced renal inflammation. This model produces CaP deposition under conditions of metabolic stress and inflammatory activation, providing a useful platform to investigate inflammation-driven mechanisms of renal mineralization.

In addition to renal CaP deposition, the biochemical profile observed in our model shares several features with previously reported hypercalciuric animal models (Letavernier et al., 2016; Bushins et al., 1995) and idiopathic calcium stone formers with Randall’s plaque (Kuo et al., 2003; Evan et al., 2003; Borofsky et al., 2019). Specifically, increased urinary calcium excretion and reduced citrate excretion were observed in the Ca and UUO + Ca groups, consistent with major lithogenic risk factors associated with CaP stone formation. In contrast, urinary oxalate and other electrolytes, including Na, K, Mg, and P, showed no marked alterations. This biochemical pattern is generally compatible with prior clinical observations suggesting that papillary plaque formation is more strongly associated with urinary calcium excretion than with oxalate or other urinary electrolytes.

Future studies incorporating anti-inflammatory and pathway-specific interventions across both the current model and genetically predisposed models, such as tissue-targeted genetic approaches (e.g., inducible or cell-type–specific modulation of MAPK, NF-κB, and Smad signaling), will be necessary to determine their causal roles in inflammation-driven renal calcification, thereby further clarifying the complex interplay between metabolic susceptibility and inflammation-associated mechanisms of crystal formation.

Osteogenesis-related proteins were shown to regulate mineralization and calcification, with OPN being a well-characterized example exhibiting a bidirectional effect on CaP crystallization. Owing to its calcium-binding properties, OPN may function as either an inhibitor or a promoter of CaP crystals depending on its local concentration and phosphorylation status (Sodek et al., 2000; Gericke et al., 2005; Iline-Vul et al., 2020). On the one hand, OPN suppresses the early stages of CaP crystallization by delaying nucleation and modulating crystal growth through interactions with free calcium ions and mineral phases (Pampena et al., 2004; de Bruyn et al., 2013). On the other hand, OPN promotes the later stages of CaP crystallization by facilitating crystal aggregation and stabilization through its incorporation into the organic matrix of calcified tissues and kidney stones (Iline-Vul et al., 2020; Sivaguru et al., 2025). This suggests that OPN functions as a stage-specific regulator, suppressing early crystal formation while facilitating later aggregation within a protein-rich matrix environment. In our experimental system, OPN showed relatively modest effects on CaP nucleation and growth but significantly enhanced crystal aggregation, supporting a context-dependent and stage-specific role of OPN in CaP crystallization.

ALP has been reported to promote calcification by hydrolyzing pyrophosphate, an inhibitor of CaP nucleation; its deficiency impairs bone mineralization, whereas overexpression contributes to vascular calcification (Lomashvili et al., 2008; Russell et al., 1971). Despite these insights, CaP stone formation is a multistep process as previously mentioned, yet most research has focused primarily on the nucleation stage. This gap is partly due to the lack of well-developed experimental models tailored to CaP, unlike those for CaOx.

Systematically, we assessed the direct effects of osteogenesis-related proteins on distinct stages of CaP crystal formation—nucleation, growth, and aggregation. While OPN specifically drove aggregation as discussed above, ALP markedly enhanced both CaP crystallization and aggregation, and OPG promoted crystal growth and aggregation (Figure 5). These distinct profiles highlight the stage-specific roles of these proteins in CaP stone pathogenesis and suggest their potential roles as modulators in nephrolithiasis. Notably, all three proteins enhanced aggregation, but OPN and OPG promoted larger aggregates (>10 crystals), indicating stronger clustering ability. Conversely, ALP favored smaller aggregates (3–5 crystals), but in greater numbers. These differences likely reflect their distinct physicochemical properties, such as structure, surface charge, and CaP binding affinity, which influence inter-crystal adhesion and aggregate formation.

Moreover, these novel non-cell-based assays for CaP offer a valuable platform for dissecting the stage-specific roles of various substances, such as proteins, toxins, or pharmacological agents, in renal calcification, and may facilitate future investigations into their contributions to crystal and stone formation.

The translocation of matrix vesicles containing needle-shaped CaP crystals from renal tubular epithelial cells to the interstitium and renal papilla has been shown to promote CaP crystal formation and Randall’s plaque development (Khan et al., 2012). Chondrocyte-derived matrix vesicles are known to carry osteogenic markers, including ALP, OPN, bone sialoprotein, osteocalcin, and osteonectin (Nahar et al., 2008). Based on these observations and our findings, we hypothesize that matrix vesicles containing osteogenesis-related proteins and calcium may bud from the basement membrane of Henle’s loop into the renal interstitium. Within these vesicles, osteogenic proteins may regulate CaP nucleation, growth, and aggregation. Matured crystals may rupture the vesicle membrane and adhere to the renal papillary surface, contributing to Randall’s plaque formation. Further studies are needed to determine whether renal tubular epithelial cell-derived vesicles indeed transport osteogenic proteins and actively participate in this pathological process.

The findings of this study may provide a basis for future clinical applications. To further validate clinical relevance, renal expression of pro-inflammatory cytokines, activation of MAPK and Smad pathways, and osteogenesis-related protein levels should be evaluated in patients with and without Randall’s plaques. Additionally, cytokine concentrations, pathway activities, and osteoblast marker expression in urine or urinary sediments should be analyzed to determine their inter-correlations and associations with plaque severity. These investigations will elucidate the molecular mechanisms of Randall’s plaque formation and explore the potential of these molecules as non-invasive biomarkers. Identifying such biomarkers could enable early detection, risk stratification, and longitudinal monitoring, ultimately improving clinical management and prevention of calcium-based kidney stone disease.

Despite these promising findings, several limitations should be acknowledged. First, while our *in vitro* results using HK-2 cells suggest the potential for osteogenic-like responses in renal tubular epithelial cells under inflammatory and high-calcium conditions, direct validation in human Randall’s plaque tissues remains lacking. Previous studies have demonstrated osteogenic marker expression in Randall’s plaque tissues and have implicated interstitial fibroblasts as a potential contributor to osteogenic signaling (Zhu et al., 2020). Accordingly, our findings do not define a direct cellular origin in human tissues, but rather suggest that multiple renal cell types may exhibit osteogenic-like phenotypes under pathological conditions. Future investigations using cell-type-specific approaches, such as co-localization immunofluorescence or single-cell RNA sequencing of human papillary tissues, will be required to define the cellular sources and functional contributions of osteogenic marker expression in Randall’s plaque formation.

Second, the experimental rat model used in this study represents an exploratory platform rather than a complete recapitulation of human Randall’s plaque. While the model successfully induces CaP deposition and osteogenic marker upregulation, the precise anatomical localization compared to human interstitial plaques remains to be fully characterized, and higher-resolution imaging approaches will be required for definitive spatial resolution. Furthermore, the limited sample size in this exploratory proof-of-concept investigation may reduce the statistical power of the quantitative analyses, and the use of young rats may not fully recapitulate age-related alterations in calcium metabolism and renal susceptibility to calcification. In addition, while local inflammatory activation and CaP deposition were confirmed within renal tissues, systemic plasma levels of inflammatory cytokines and vitamin D were not evaluated. Therefore, future studies incorporating larger cohorts, mature animal models, and systemic inflammatory and metabolic profiling will be required to further strengthen the translational relevance of this model and clarify the interplay between systemic and local mechanisms in early inflammation-driven renal calcification.

Beyond the kidney, the inflammatory–osteogenic axis observed in this study may represent a conserved mechanism driving pathological mineralization across diverse organ systems. For example, fibroproliferative conditions such as fibrodysplasia ossificans progressiva involve inflammatory cues that drive soft tissue calcification and upregulation of osteogenic markers like Runx2 and ALP (Micha et al., 2016). Similarly, cartilage calcification in osteoarthritis is associated with inflammation and activation of osteogenic programs in chondrocytes (Richter et al., 2023; Bernabei et al., 2023). Tumor-associated microcalcifications in breast (Oyama et al., 2002) and thyroid tissue (Ferreira et al., 2018; Sun et al., 2011) also show elevated osteopontin expression in calcified areas, indicating involvement of osteogenic protein activity. The inflammatory–osteogenic axis observed in this study may represent a conserved mechanism driving pathological mineralization across organ systems. Future studies should investigate whether this shared pathway underlies other forms of ectopic ossification, thereby extending the relevance of our renal model to broader human disease contexts.

**FIGURE 9:**
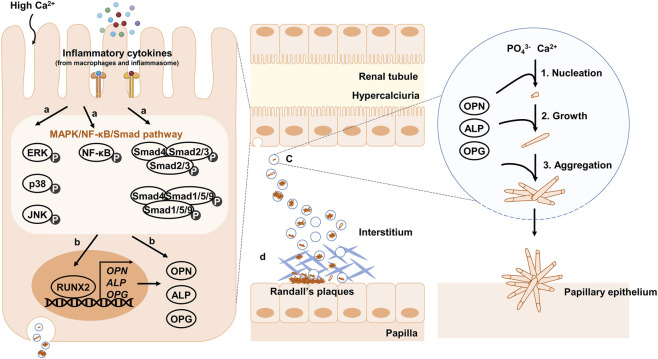
Hypothesized Model of Randall’s Plaque Formation. a, In a high-calcium renal environment, activated macrophages and inflammasomes release pro-inflammatory cytokines, activating signaling pathways such as MAPK, NF-κB, and Smad in renal tubular cells. b, These signaling pathways upregulate proteins involved in osteogenesis, such as Runx2, ALP, OPG, and OPN, and result in an osteogenic transition. c, Calcium-enriched and osteogenesis-related protein-enriched vesicles are released from the basal side of tubule epithelial cells. Osteogenesis-related proteins directly promote the nucleation, growth and aggregation of CaP crystals in these vesicles. d, CaP aggregates-enriched vesicles move to the renal interstitium, calcifying the interstitial collagen, and eventually leading to the formation of Randall’s plaque in the renal papillae. This figure was created by BioRender.com.

In summary, our results indicate that pro-inflammatory cytokines may enhance calcium-induced renal calcification by upregulating osteogenesis-related proteins through the MAPK, NF-κB, and Smad signaling pathways. These proteins directly regulate key stages of CaP crystal formation, including nucleation, growth, and aggregation (Figure 9). Collectively, these signaling pathways may converge to integrate inflammatory and calcium-mediated stress signals, thereby promoting osteogenic-like changes of renal tubular cells and subsequent CaP deposition. Beyond identifying potential therapeutic targets for calcium-based kidney stone prevention, we also established a novel short-term rat model of renal CaP deposition, which may serve as a practical platform for investigating mechanisms of renal calcification, including processes potentially relevant to Randall’s plaque–associated calcification.

## Data Availability

The raw data supporting the conclusions of this article will be made available by the authors, without undue reservation.
