# Supplementary material for: Inflammation-induced osteogenic signaling promotes calcium phosphate crystal formation in kidneys via MAPK, NF-κB, and smad pathways
Source: Front Cell Dev Biol. 2026 Jun 30;14:1831072. doi: 10.3389/fcell.2026.1831072 (PMC13365047; doi:10.3389/fcell.2026.1831072)
Supplement: Supplementary file 1 [file DataSheet1.docx]

Supplementary Material

## Supplementary Figures


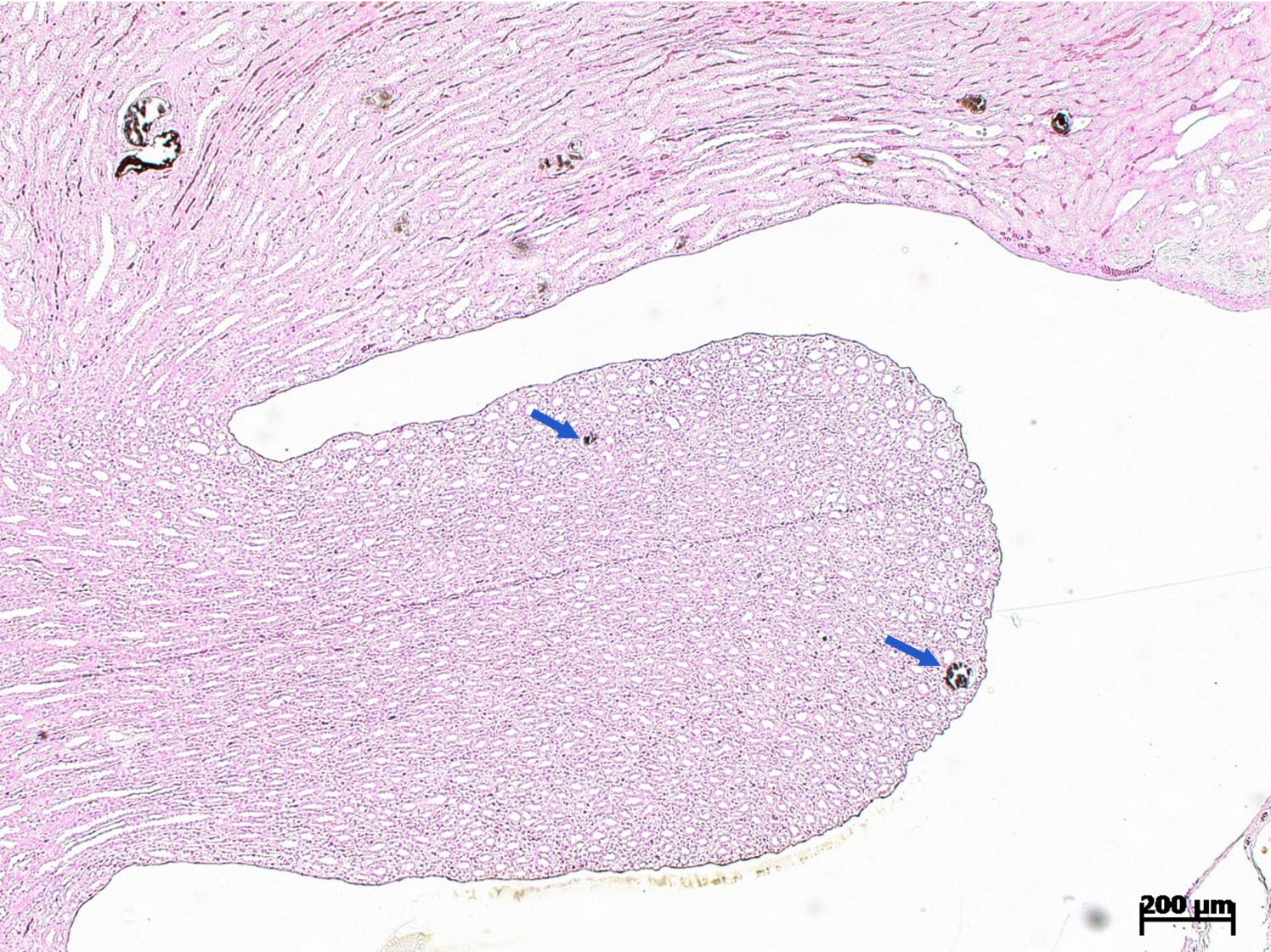


**Supplementary Figure 1. Representative histological images illustrating calcium phosphate deposition in the renal papillary region.** Representative histological image of a rat kidney section from the UUO+Ca group stained with Von Kossa (50× magnification). Blue arrows indicate Von Kossa-positive calcium phosphate (CaP) deposits within the structural confines of the renal papilla. Scale bar, 200 μm.
